# Primary Amenorrhea: Gonadal Dysgenesis and Primary Ovarian Insufficiency—Distinct Entities or a Continuum?

**DOI:** 10.5812/ijem-172709

**Published:** 2026-01-31

**Authors:** Kyana Jafarabady, Fahimeh Ramezani Tehrani

**Affiliations:** 1 Reproductive Endocrinology Research Center, Research Institute for Endocrine Molecular Biology, Research Institute for Endocrine Sciences, Shahid Beheshti University of Medical Sciences, Tehran, Iran; 2 Foundation for Research and Education Excellence, Vestavia Hills, Al, USA

**Keywords:** Primary, Ovarian, Insufficiency

An 18-year-old woman presented with primary amenorrhea following an initial evaluation at 16 years of age for delayed pubertal development. Laboratory studies revealed markedly elevated gonadotropin levels (LH, 44.5 IU/L; FSH, 46.6 IU/L) and absent secondary sexual characteristics (Tanner stage B1, PH1). Pelvic ultrasonography demonstrated a hypoplastic uterus with nonvisualized, atrophic ovaries. Karyotype analysis showed a normal 46, XX pattern. Hormone replacement therapy (HRT) was initiated, and after 2 years, imaging revealed normalization of ovarian size and progression of secondary sexual characteristics (Tanner stage B4, PH3).

This case highlights a persistent clinical dilemma: distinguishing primary ovarian insufficiency (POI) from 46, XX gonadal dysgenesis in young women presenting with hypergonadotropic hypogonadism. In patients with primary amenorrhea, elevated gonadotropin levels, and a normal 46, XX karyotype, these entities may initially appear indistinguishable. However, they differ fundamentally in pathophysiology, prognosis, and reproductive potential (1, 2). This lack of precise stratification has practical implications, particularly as therapeutic options expand.

Etymologically, dysgenesis derives from the Greek dys- (abnormal) and -genesis (development), denoting defective organ formation. In 46, XX gonadal dysgenesis, ovarian failure is congenital and characterized by streak gonads and absent follicular development. Estrogen exposure is minimal, and spontaneous pubertal progression does not occur. In contrast, POI involves structurally formed ovaries with premature follicular depletion or dysfunction. Many affected individuals experience a period of normal ovarian activity, permitting partial or complete pubertal maturation before ovarian decline. Despite these biological distinctions, both conditions remain grouped under the umbrella of hypergonadotropic hypogonadism. This broad classification may obscure clinically meaningful differences and underscores the need for more refined diagnostic frameworks (3).

In POI, estrogen therapy may stimulate ovarian enlargement and reveal residual follicular tissue, as demonstrated in this case. In gonadal dysgenesis, the absence of viable follicles renders the gonads unresponsive to hormonal stimulation. Thus, treatment response may serve as a valuable adjunctive diagnostic tool. The ovarian enlargement observed following HRT in this patient strongly supports residual ovarian function and favors a diagnosis of POI. The importance of accurate differentiation is further magnified by the emergence of regenerative reproductive interventions, including platelet-rich plasma (PRP) and stem cell–based therapies, which have shown encouraging results in selected patients with POI (4).

Such approaches rely on residual follicular activity and would be ineffective in true gonadal dysgenesis, in which viable ovarian tissue is absent. Precise classification, therefore, is no longer merely semantic; it directly influences therapeutic eligibility and reproductive counseling.

As reproductive medicine enters an era of regenerative possibilities, clearer diagnostic stratification between POI and 46, XX gonadal dysgenesis is imperative. Refining guideline criteria to incorporate longitudinal clinical assessment, imaging evolution, and therapeutic response may improve diagnostic accuracy. Ultimately, accurate distinction enhances prognostic clarity, informs treatment selection, and ensures that emerging fertility-preserving interventions are appropriately targeted.
